# LINC00665 regulates hepatocellular carcinoma by modulating mRNA via the m6A enzyme

**DOI:** 10.1515/biol-2022-0003

**Published:** 2022-02-11

**Authors:** Ming Lei, Xinghua Du, Xiaokai Li, Fuke Wang, Ling Gu, Feng Guo

**Affiliations:** Nursing Health Sciences College, Yunnan Open University, Kunming, Yunnan, 650500, China; Laboratory Medicine Department, The Integrated Traditional Chinese and Western Medicine Hospital of Yunnan Province, Kunming, Yunnan, 650224, China; Hepatobiliary Surgery Department, The First Affiliated Hospital of Kunming Medical University, Kunming, Yunnan, 650032, China; Sport Medicine Department, The First Affiliated Hospital of Kunming Medical University, Kunming, Yunnan, 650032, China; Pain Department, The First Affiliated Hospital of Kunming Medical University, Kunming, Yunnan, 650032, China; The Clinical Skills Training Center, Kunming Medical University, No. 1168 Chunrongxi Road Chenggong District, Kunming, Yunnan, 650500, China

**Keywords:** hepatocellular carcinoma, N6-methyladenosine, long noncoding RNA, prognosis, comprehensive network

## Abstract

This study aimed to reveal the mechanism by which long noncoding RNAs (lncRNAs) modulate hepatocellular carcinoma (HCC) by regulating mRNA via the N6-methyladenosine (m6A) enzyme. The expression and clinical data of 365 HCC patients and 50 healthy control samples were downloaded from the the Cancer Genome Atlas (TCGA) database. Differentially expressed lncRNAs (DElncRNAs) and differentially expressed mRNAs (DEmRNAs) screened using limma packages from the R. m6A2Target database were used to predict the relationship between m6A enzyme-lncRNA and m6A enzyme-mRNA. The mRNAs in the lncRNA-m6A enzyme-mRNA network were subjected to enrichment analysis. Cox regression analysis was used to screen for RNAs significantly related to HCC prognosis. The expression of differentially expressed RNAs (DERs) was verified using the TCGA dataset and GSE55092. Eighty-five DElncRNAs and 747 DEmRNAs were identified. The mRNAs in the lncRNA-m6A enzyme-mRNA network were primarily involved in mitotic cell division, the p53 signaling pathway, and the cell cycle. Three lncRNAs and 14 mRNAs were significantly associated with HCC prognosis. Furthermore, the expression of 12 DERs differed significantly between patients in the early and advanced stages. LINC00665 was predicted to regulate 11 mRNAs by modulating IGF2BP1, IGF2BP2, and YTHDF1. Thus, this study provides new insights into the roles of lncRNA and m6A enzymes in HCC.

## 1 Introduction

Hepatocellular carcinoma (HCC) is the third leading cause of cancer-related deaths worldwide [1]. HCC usually occurs in the context of chronic liver disease, and its occurrence is related to complex interactions among the host, the disease, and environmental factors. Among them, chronic hepatitis B or C virus infection is the primary risk factor for HCC worldwide [2]. Histological examination of the liver has consistently been used as the gold standard for the diagnosis of HCC. However, the risk of bleeding and tumor cell proliferation during biopsy is an ongoing problem during diagnosis [3]. Statistically, the 5-year survival rate of primary liver cancer in the United States is approximately 16% [4], which is close to that in Europe [5], whereas the survival rate of HCC in underdeveloped areas may be less than 5%. Thus, there is considerable research being undertaken currently on understanding the molecular genetic mechanism underlying HCC to provide a theoretical basis for the diagnosis and treatment of HCC.

N6-methyladenosine (m6A) accounts for approximately 50% of ribonucleotides in mammals [6]. Similar to DNA methylation, epigenetic regulation of m6A modulation is reversible in mammalian cells. The m6A enzymes include “writers,” “erasers,” and “readers,” which can add, remove, or recognize m6A-modified sites, respectively, to alter important biological processes [7]. M6A methylation affects RNA expression, shearing, and translation. Various studies have found that m6A methylation plays a critical role in tumor progression [8]. In addition, abnormal m6A modifications are closely associated with cancer. Researchers have observed that the methylation of m6A RNA is associated with the self-renewal of glioblastoma stem cells (GSCs), and the presence of GSCs indicates a poor prognosis for glioblastoma multiforme [9]. The m6A methylation enzyme, methyltransferase-like 3 (METTL3), participates in maintaining pluripotency and inhibiting cell differentiation in acute myeloid leukemia by mediating the elevation of m6A [10]. Abnormal upregulation of METTL3 has been identified in patients with HCC, and it often involves the progression of HCC.

Long noncoding RNAs (lncRNAs) are functionally diverse species of noncoding RNA. Studies have demonstrated that lncRNA expression may be closely related to cancer development [11,12]. Several studies have elucidated the regulatory effects of lncRNAs on HCC. Wang et al. reported that lncRNA MCM3AP-AS1 promotes the growth of HCC cells [13], whereas Chen et al. identified that lncRNA CDKN2BAS could predict poor prognosis in patients with HCC and promote metastasis [14]. However, few studies have delineated the exact mechanism by which lncRNAs regulate mRNA expression by modulating m6A regulatory factors.

Based on The Cancer Genome Atlas (TCGA), RNA-seq expression dataset, and m6A enzyme-targeting gene database, this study aimed to reveal how lncRNAs play a role in HCC by regulating m6A regulators to further influence mRNA (Figure S1).

## 2 Materials and methods

### 2.1 Data collection and preprocessing

HCC gene expression data were downloaded from the TACG database (https://gdc-portal.nci.nih.gov/) on April 28, 2021. A total of 423 samples were included, comprising 365 tumor samples with clinical information and 50 healthy control samples. The detected lncRNAs and mRNAs were annotated according to the annotation information of the detection platform (Illumina HiSeq 2000 RNA Sequencing, Illumina, Inc., San Diego, California).

### 2.2 Screening of differentially expressed RNAs (DERs)

Samples were divided into tumor and control groups based on the sample information. DERs between the tumor and control groups were screened using the limma package (version 3.347, Walter and Eliza Hall Institute of Medical Research in Melbourne, Australia) of R3.6.1 [15] with a false discovery rate (FDR) of <0.05, and |log 2 fold change| >1 as the threshold for significance. Subsequently, a bidirectional hierarchical clustering based on the centered Pearson correlation algorithm using R3.6.1 pheatmap version 1.2.8 [16] was performed on the expression values of DERs.

### 2.3 Comprehensive network construction and enrichment analysis

First, the m6A2Target database [17] was used to predict the lncRNAs and mRNAs related to the m6A enzyme. Second, the m6A-related lncRNAs and mRNAs intersected with the significant DERs screened in the previous step. Subsequently, these intersected DERs were selected to construct the differentially expressed lncRNAs (DElncRNAs)-m6A enzyme and differentially expressed mRNAs (DEmRNAs)-m6A enzyme network. After that, the Pearson correlation coefficient (PCC) between the expression levels of significant DElncRNAs and DEmRNAs related to the m6A enzyme was calculated. The DElncRNAs–DEmRNAs coexpression network was constructed with *p* <0.05 and |PCC| >0.5. Furthermore, the lncRNAs-m6A enzyme-mRNAs comprehensive network was constructed by combining the DElncRNAs-m6A enzyme and DEmRNAs-m6A enzyme network. All the networks constructed in this study were visualized using Cytoscape (version 3.6.1, National Institute of General Medical Sciences, USA) software (http://www.cytoscape.org/).

Finally, significant DEmRNAs in the comprehensive network underwent gene ontology (GO) and Kyoto Encyclopedia of Genes and Genomes (KEGG) analyses using DAVID (version 6.8, Laboratory of Human Retrovirology and Immunoinformatic, USA) [18,19]. Terms with more than two genes enriched and FDR <0.05 were considered to be significantly enriched terms.

### 2.4 Prognostic correlation analysis of lncRNAs and mRNAs in the comprehensive network

Univariate and multivariate Cox regression analyses in the survival package (version 2.41-1) [20] of R3.6.1 were used to screen for significantly independent prognostic lncRNAs and mRNAs. Based on the median expression level of target lncRNAs and mRNAs, the samples were divided into groups with a high expression level (expression level above or equal to the median expression level) and low expression level (expression level below the median expression level). The Kaplan–Meier (K–M) curve was used to evaluate the association between target lncRNAs and mRNA expression levels as well as prognosis.

### 2.5 Expression level analysis of lncRNAs and mRNAs in the comprehensive network in different clinical groups

Independent prognostic clinical factors were screened using univariate and multivariate Cox regression analyses in the survival package (version 2.41-1) of R3.6.1. A log-rank *p* value less than 0.05 was selected as the threshold for screening independent prognostic clinical factors.

Based on the screened independent prognostic clinical factor information, the samples were divided into different clinical factor feature groups, and the variations in the expression levels of the DERs screened in the previous step were analyzed using the between groups *t*-test in R3.6.1.

### 2.6 Verification of the expression of DERs

The expression values of RNAs with significantly different expression levels in different independent prognostic clinical factors screened in the previous step were extracted from the TCGA dataset to analyze the expression differences of these RNAs between tumor samples and normal samples. GSE55092 was subsequently downloaded from the National Center for Biotechnology Information Search database Gene Expression Omnibus database to analyze the expression of DERs. A total of 49 HCC tumor samples and 91 control samples were included in the GSE55092.

## 3 Results

### 3.1 Screening of DERs

**Figure 1:**
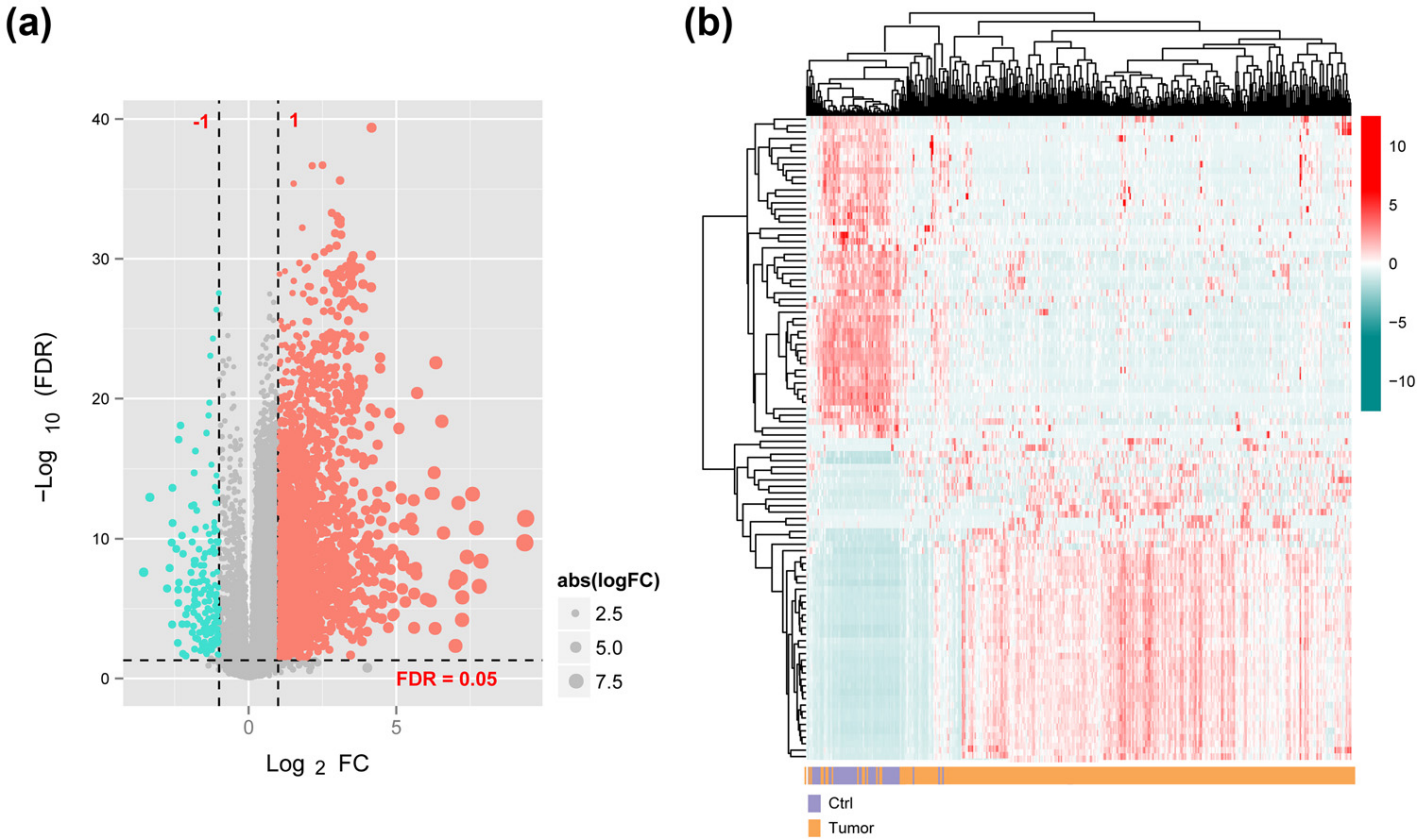
DElncRNAs and DEmRNAs (DERs): (a) the volcano plot of DERs and (b) two-way hierarchical clustering heat map of top50 expression levels of DERs. Red and cyan dots indicate significant differences between upregulated and downregulated expressed DERs, respectively.

After annotation, 1,241 lncRNAs and 14,164 mRNAs were identified. A total of 85 DElncRNAs and 747 DEmRNAs were screened between the tumor and control groups (Figure 1a). Thereafter, the upregulated and downregulated top 50 DERs were selected for bidirectional hierarchical clustering according to the ascending power of their log FC values. The heat map obtained has been depicted in Figure 1b, wherein it can be noted that the samples were aggregated in two different directions.

### 3.2 Comprehensive network construction

**Figure 2:**
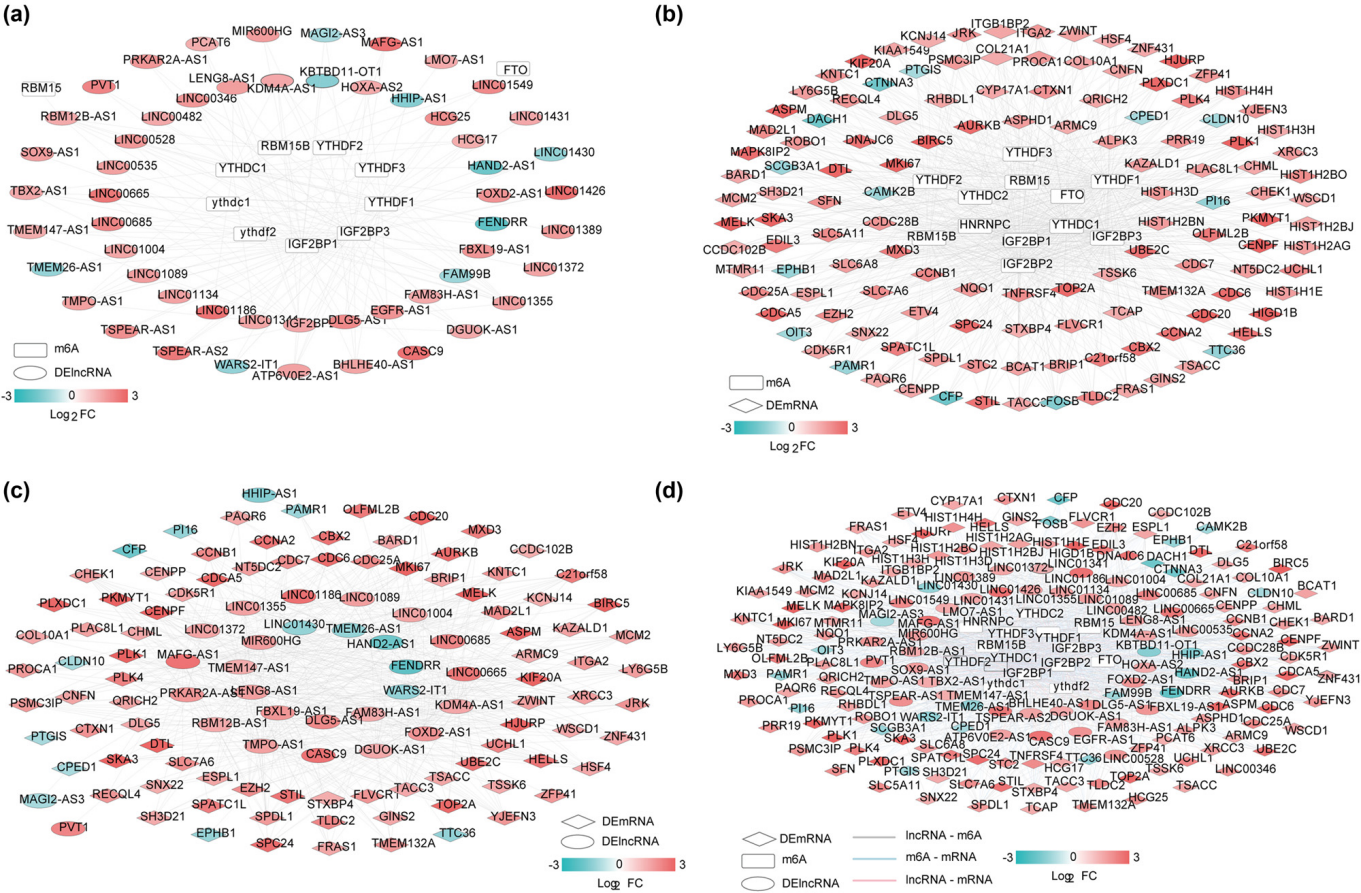
(a) lncRNA-m6A protein network, (b) mRNA-m6A enzyme network, (c) mRNA-lncRNA network, and (d) lncRNA-m6A enzyme-mRNA comprehensive network.

m6A enzyme-related lncRNAs were intersected with DElncRNAs, following which 53 DElncRNAs related to m6A enzyme and 12 m6A enzyme enzymes were identified (Figure 2a). This network included 162 connection pairs. Furthermore, a DEmRNAs-m6A enzyme network was also constructed, comprising 127 m6A enzyme-related DEmRNAs and 14 m6A enzyme enzymes (Figure 2b). Subsequently, the lncRNA-mRNA coexpression network containing 53 DElncRNAs and 127 DEmRNAs was created (Figure 2c). There were 945 significantly correlated connection pairs identified in this coexpression network.

In conjunction with the results of the above analysis, a DElncRNAs-m6A enzyme-DEmRNAs network was constructed (Figure 2d). Moreover, the enrichment analysis of GO and KEGG signaling pathways was performed on the DEmRNAs that constituted the DElncRNAs-m6A enzyme-DEmRNAs network. A total of 16 significant GO biological processes and 7 KEGG signaling pathways were screened (Table 1). Enriched biological processes primarily included cell division, proliferation, and mitotic division. Similarly, the KEGG pathways are generally involved in transcriptional misregulation in cancer, the FoxO signaling pathway, the p53 signaling pathway, and cell cycle.

**Table 1:** GO biological process and KEGG signal pathways in which mRNAs in the comprehensive network are significantly related

Category	Term	Count	*p* Value	FDR
Biology process	GO:0007062∼sister chromatid cohesion	12	3.35 × 10^−11^	1.40 × 10^−8^
	GO:0051301∼cell division	18	4.06 × 10^−11^	1.40 × 10^−8^
	GO:0007067∼mitotic nuclear division	15	3.49 × 10^−10^	8.02 × 10^−8^
	GO:0006260∼DNA replication	10	4.62 × 10^−7^	7.97 × 10^−5^
	GO:0008283∼cell proliferation	13	2.47 × 10^−6^	3.41 × 10^−4^
	GO:0006334∼nucleosome assembly	8	8.49 × 10^−6^	8.37 × 10^−4^
	GO:0000086∼G2/M transition of mitotic cell cycle	8	2.13 × 10^−5^	1.84 × 10^−3^
	GO:0000082∼G1/S transition of mitotic cell cycle	7	3.85 × 10^−5^	2.95 × 10^−3^
	GO:0031145∼anaphase-promoting complex-dependent catabolic process	6	1.24 × 10^−4^	8.54 × 10^−3^
	GO:0031577∼spindle checkpoint	3	2.22 × 10^−4^	1.39 × 10^−2^
	GO:0000070∼mitotic sister chromatid segregation	4	4.67 × 10^−4^	2.48 × 10^−2^
	GO:0031536∼positive regulation of exit from mitosis	3	5.50 × 10^−4^	2.71 × 10^−2^
	GO:0007093∼mitotic cell cycle checkpoint	4	9.77 × 10^−4^	4.29 × 10^−2^
	GO:0051726∼regulation of cell cycle	6	9.95 × 10^−4^	4.29 × 10^−2^
	GO:1904668∼positive regulation of ubiquitin protein ligase activity	3	1.30 × 10^−3^	4.74 × 10^−2^
	GO:0030071∼regulation of mitotic metaphase/anaphase transition	3	1.30 × 10^−3^	4.74 × 10^−2^
KEGG pathway	hsa04110:Cell cycle	13	3.42 × 10^−14^	2.77 × 10^−12^
	hsa04114:Oocyte meiosis	7	1.35 × 10^−7^	1.09 × 10^−5^
	hsa04914:Progesterone-mediated oocyte maturation	6	3.97 × 10^−7^	3.22 × 10^−5^
	hsa04115:p53 signaling pathway	3	9.78 × 10^−5^	7.92 × 10^−3^
	hsa04068:FoxO signaling pathway	3	2.96 × 10^−4^	2.40 × 10^−2^
	hsa05202:Transcriptional misregulation in cancer	3	4.02 × 10^−4^	3.26 × 10^−2^
	hsa04974:Protein digestion and absorption	2	5.71 × 10^−4^	4.62 × 10^−2^

### 3.3 Prognostic correlation analysis

**Figure 3:**
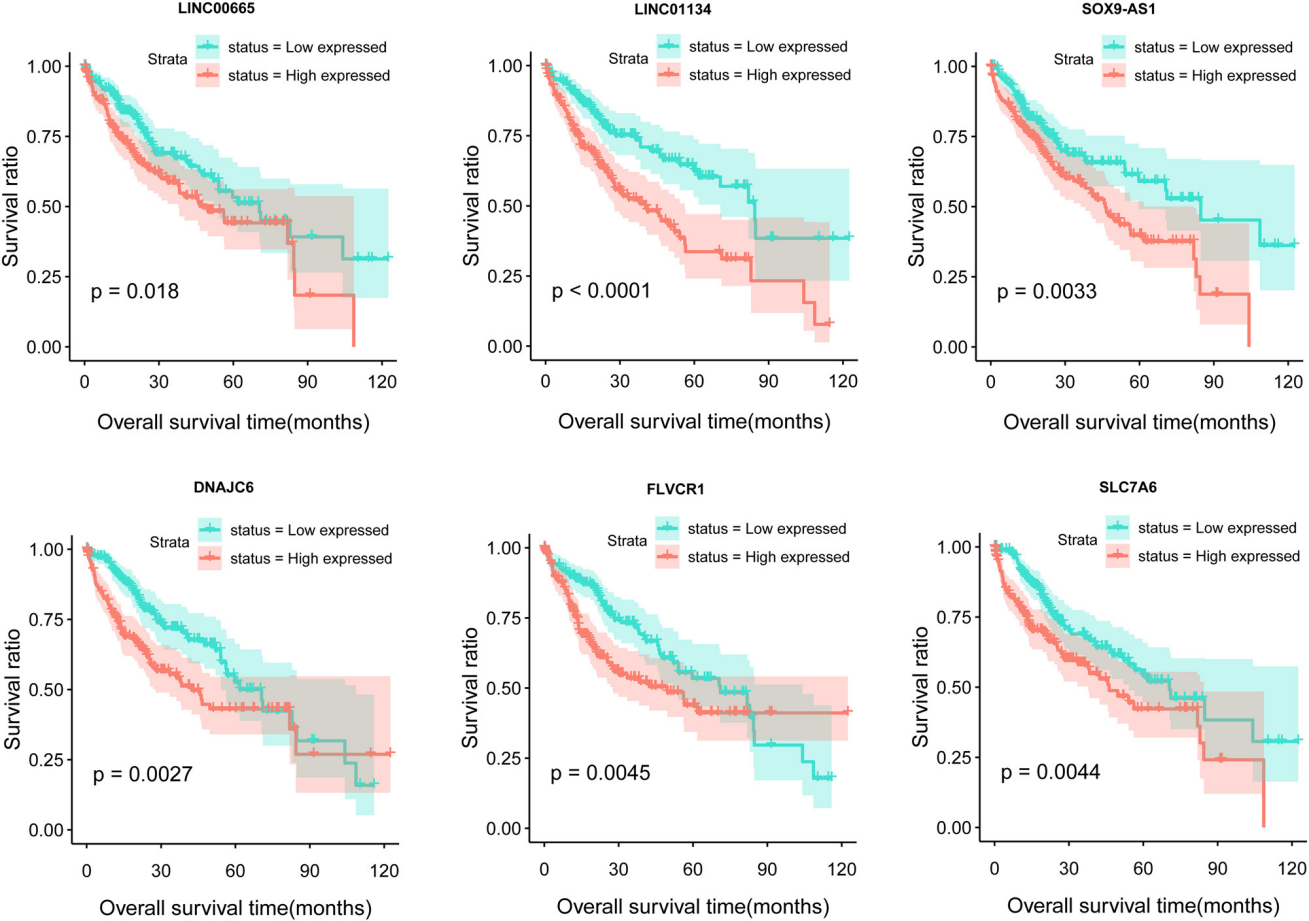
The K–M curve of the lncRNAs and mRNAs that are significantly correlated with independent prognosis.

From the DElncRNAs and DEmRNAs included in the comprehensive network – along with clinical prognosis information of TCGA samples – univariate Cox regression analysis was used to screen the DERs significantly associated with survival prognosis. A total of 83 DERs significantly associated with prognosis were screened, including 7 lncRNAs and 76 mRNAs (Table S1). Multivariate Cox regression analysis was subsequently performed on these 83 DERs for independent prognostic-related DERs. Finally, 17 significantly independent prognosis-related DERs were obtained (Table 2). Patients were divided into two groups according to the median expression level of the 17 DERs. The K–M analysis revealed that the low expression of these DERs resulted in a better prognosis (*p* < 0.05; Figure 3). The other results have been presented in Figure S2.

**Table 2:** RNAs that were significantly associated with prognosis using multivariate Cox regression analysis

ID	Type	Coef	Hazard ratio	*z*	Pr(>|*z*|)
SOX9-AS1	lncRNA	0.7263754	2.0675728	3.181	0.001466
LINC00665	lncRNA	−0.3784111	0.6849489	−1.996	0.04588
LINC01134	lncRNA	0.8595095	2.362002	1.713	0.0486715
FLVCR1	mRNA	1.2147971	3.3696102	3.769	0.000164
DNAJC6	mRNA	1.0555246	2.8734821	3.623	0.000292
SLC7A6	mRNA	−1.6519996	0.1916663	−3.529	0.000417
ZWINT	mRNA	1.3523435	3.8664762	2.928	0.003416
KIF20A	mRNA	1.5733317	4.8226893	2.864	0.004186
CENPF	mRNA	−1.3804044	0.2514768	−2.507	0.012171
CCNB1	mRNA	−0.9717552	0.3784183	−2.175	0.029622
CBX2	mRNA	0.4877579	1.6286604	2.122	0.03384
NQO1	mRNA	0.1078229	1.1138504	2.076	0.037923
SFN	mRNA	0.1605477	1.1741538	2.072	0.038287
CCNA2	mRNA	0.4334967	1.5426422	2.021	0.043259
FRAS1	mRNA	0.5390393	1.7143591	1.994	0.046132
SPC24	mRNA	−0.5799142	0.5599464	−1.992	0.046388
RECQL4	mRNA	−0.6959589	0.4985961	−1.989	0.046662

### 3.4 The expression level of DERs in different clinical groups within the integrated network

**Figure 4:**
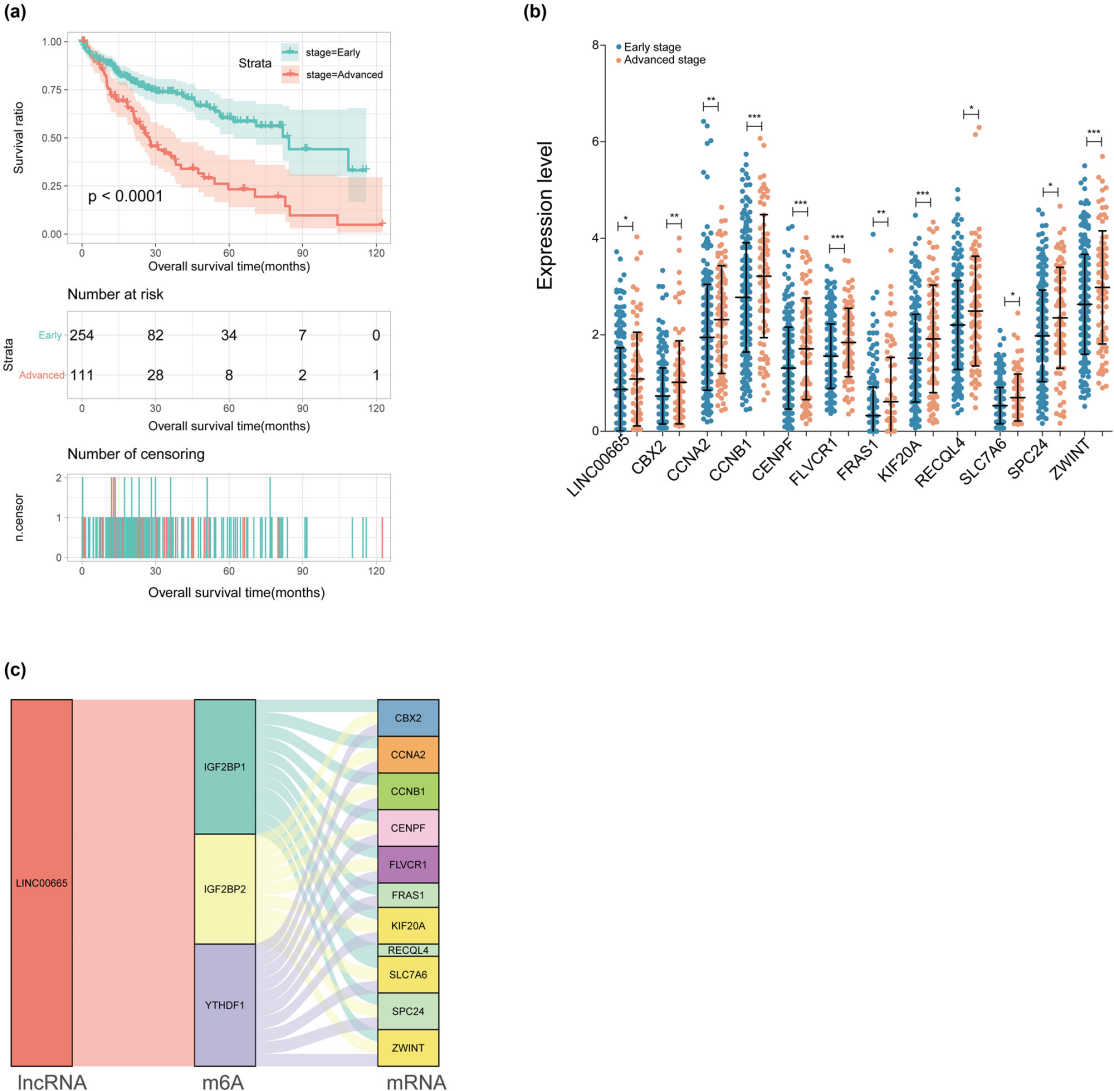
(a) The prognostic K–M curve of patients with different pathologic stages, (b) 12 RNAs expression levels with significant differences in patients of various pathologic stages. (c) Sankey diagram containing lncRNA, m6A enzymes, and mRNAs.

Univariate and multivariate Cox regression analyses revealed that the pathologic stage was an independent prognostic clinical factor (Table 3). Consequently, the samples were divided into early (stage 1–2) and advanced (stage 3–4). The K–M curve revealed that patients in the early group had a significantly better prognosis than those in the advanced group (Figure 4a).

**Table 3:** Univariate and multivariate Cox regression analysis identified independent prognostic clinical factor

Clinical characteristics	Univariate Cox		Multivariate Cox	
	HR (95% CI)	*p* value	HR (95% CI)	*p* value
Age (years, mean ± SD)	1.012 (0.998–1.026)	4.790 × 10^−2^	1.011 (0.997–1.026)	1.270 × 10^−1^
Gender (men/women)	0.817 (0.573–1.164)	2.618 × 10^−1^	—	—
Pathologic M (M0/M1/–)	4.032 (0.763–12.83)	1.057 × 10^−1^	—	—
Pathologic N (N0/N1/–)	2.004 (0.491–8.181)	3.327 × 10^−1^	—	—
Pathologic T (T1/T2/T3/T4/–)	1.675 (0.916–2.007)	1.017 × 10^−1^	—	—
Pathologic stage (I/II/III/IV/–)	1.661 (1.355–2.037)	1.034 × 10^−6^	1.668 (1.359–2.045)	9.240 × 10^−7^
Histologic grade (G1/G2/G3/G4)	1.121 (0.887–1.416)	3.392 × 10^−1^	—	—
Vascular invasion (Yes/No/–)	1.351 (0.892–2.047)	1.537 × 10^−1^	—	—
Recurrence (Yes/No/–)	1.375 (0.914–2.068)	1.249 × 10^−1^	—	—

In addition, the expression of 17 DERs between patients in the early and advanced groups was also analyzed. The results indicated that 12 of these DERs exhibited a significantly higher expression level in the advanced group than in the early group (*p* < 0.05) – including LINC00665, chromobox 2 (CBX2), cyclin A2 (CCNA2), cyclin B1 (CCNB1), centromere protein F (CENPF), FLVCR heme transporter 1 (FLVCR1), Fraser extracellular matrix complex subunit 1 (FRAS1), kinesin family member 20A (KIF20A), recQ like helicase 4 (RECQL4), solute carrier family 7 member 6 (SLC7A6), SPC24 component of NDC80 kinetochore complex (SPC24), and ZW10 interacting kinetochore protein (ZWINT; Figure 4b). The Sankey diagram revealed that LINC00665 was modified by the m6A enzymes IGF2BP1, IGF2BP2, and YTHDF1. Moreover, these three m6A enzymes regulated the methylation of the 11 mRNAs (Figure 4c).

Furthermore, based on the m6A2Target database, we counted the evidence for binding 11 mRNAs, one lncRNA, and three m6A enzymes involved in Figure 4c (Table S2). The results revealed that in the Huh-7 cell line, once *IGF2BP1* was knocked down, the expression levels of LINC00665 and ZWINT will be upregulated and downregulated, respectively. In addition, several studies have confirmed a direct interaction between the m6A enzyme and mRNA using eCLIP-seq.

### 3.5 Verification of the expression of DERs

**Figure 5:**
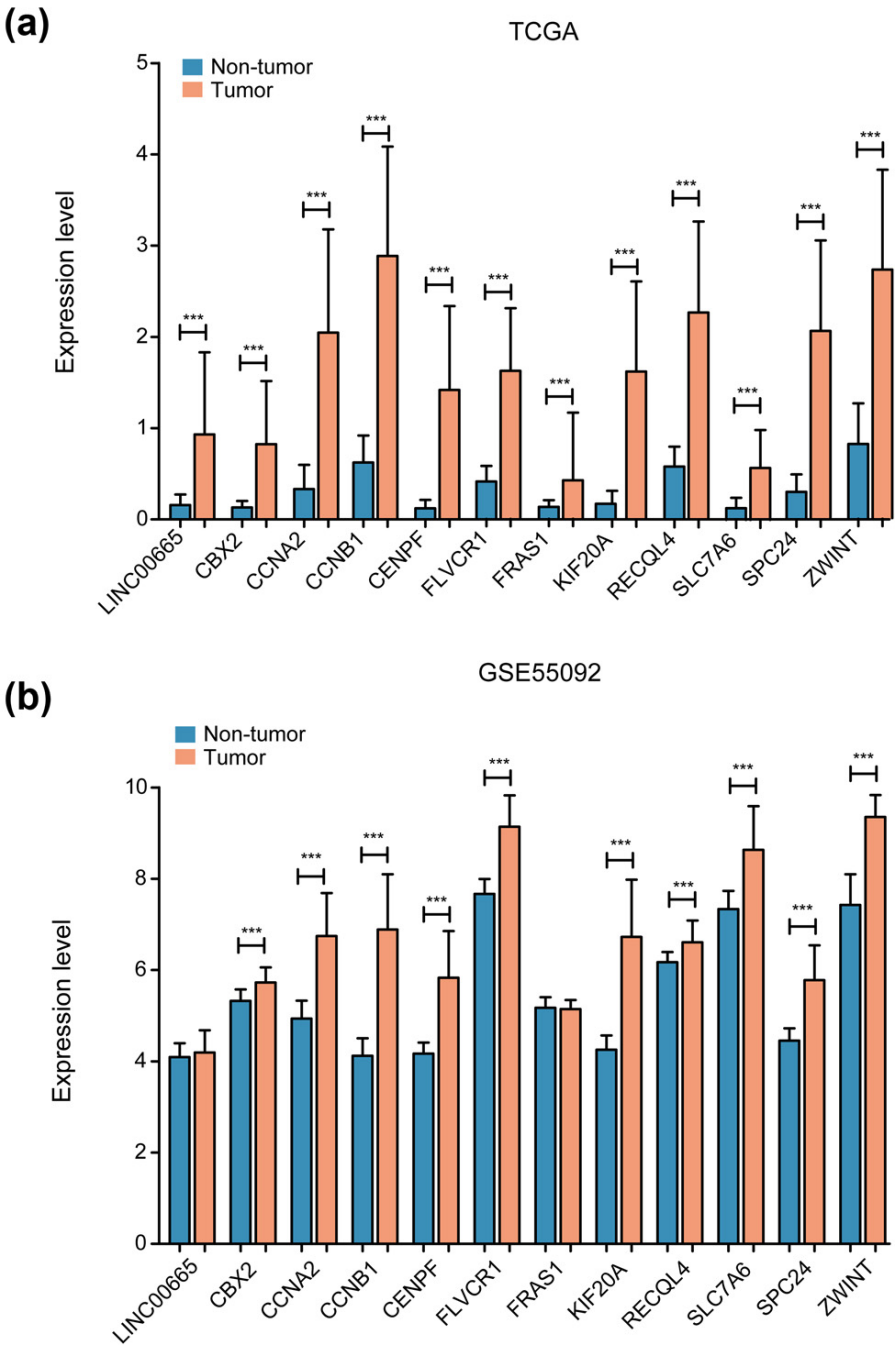
The relative expression levels of 12 RNAs in (a) TCGA and (b) GSE55092 database between tumor and control samples.

To analyze the expression of 12 DERs between tumor samples and normal samples, the expression values of these 12 DERs were extracted from the TCGA database. The results indicated that these 12 DERs exhibited a significantly higher expression in the tumor samples than in the normal samples (*p* < 0.001; Figure 5a). GSE55092 was used to validate the expression of 12 DERs. The results revealed that, except for LINC00665 and *FRAS1*, all the other 10 mRNAs exhibited significantly higher expression in tumor samples than in normal samples (*p* < 0.001; Figure 5b).

## 4 Discussion

To understand how lncRNAs play a role in HCC by regulating mRNA expression via the modulation of m6A enzymes, 85 and 747 DElncRNAs and DEmRNAs were screened, respectively. A comprehensive network containing m6A proteins, lncRNAs, and mRNAs was constructed. The mRNAs included in this network were primarily involved in the mitotic division, cell cycle checkpoints, and the p53 signaling pathway. It is well established that mitotic cell division is a series of processes designed to accurately propagate the genomic material from a maternal cell into two daughter cells. This process is important for protecting genome integrity [21]. Studies have reported that any disorder in the process of cell division may lead to malignancy [22]. The p53 tumor suppressor gene mutated in more than half of all human malignancies plays a critical role in regulating the cell cycle, cell senescence, and apoptosis and maintaining genomic stability [23]. In addition, there are several reports on the role of p53 in HCC [24].

Following the prognostic and Cox regression analyses, 3 lncRNAs and 14 mRNAs were identified as independent prognostic RNAs. Low expression of these 17 RNAs in TCGA samples was significantly associated with a better prognosis. The samples were further divided into early and advanced stages according to the independent prognostic clinical factor of the pathologic stage, following which 12 RNAs (1 lncRNA and 11 mRNAs) were screened and observed to be expressed significantly differently in various groups. LINC00665 plays a role in cancer development. An earlier study has reported that LINC00665 may be involved in HCC cell cycle regulation, and the high expression of LINC00665 in HCC patients is significantly associated with poor prognosis [25]. This finding is consistent with our observations. Further *in vitro* experiments confirmed that the depletion of LINC00665 inhibits HCC cell viability and induces cell apoptosis as well as autophagy [26]. However, there have been no studies related to LINC00665 and m6A.

Based on our hypothesis, LINC00665 could regulate 11 mRNAs by modulating the m6A enzymes IGF2BP1, IGF2BP2, and YTHDF1. In lung adenocarcinoma tissues, the expression of LINC00665 is closely related to the aggressive clinicopathological characteristics of patients and can be used as an independent predictor of relapse-free survival [27]. CIP2A-BP is a micropeptide encoded by LINC00665, and its expression in breast cancer cell lines can be downregulated by TGF-b. In breast cancer, downregulated CIP2A-BP expression is associated with tumor metastasis and poor overall survival [28]. It has been earlier reported that LINC00665 can also regulate the occurrence and development of HCC by regulating the expression of cell cycle-related genes *CCNA2* and *CCNB1* [25]. Further research is necessary to verify whether LINC00665 affects the expression of *CCNA2* and *CCNB1* via the m6A enzyme. The protein encoded by *CCNA2* is a cell cycle regulator [29]. *CCNA2* has been proven to be a target of miRNAs that regulate the proliferation of HCC cells [30]. Moreover, as a key promoter of mitosis control, the role of CCNB1 in HCC has also been widely reported. The high expression of CCNB1 is usually closely related to the poor prognosis of HCC patients [31,32]. In this study, the expression of CCNB1 in patients with advanced-stage HCC was significantly higher than that in patients with early stage. It has been reported that CBX2 could regulate proliferation and apoptosis via the phosphorylation of YAP in HCC [33]. High expression of FLVCR1 was significantly associated with poor prognostic value in HCC patients [34]. *CENPF* was identified as a potential prognostic biomarker and target for HCC [35]. It is reported that upregulated *RECQL4* [*36*], *SPC24* [37], and *KIF20A* [38] could predict poor prognosis in HCC, whereas upregulated *ZWINT was* associated with great prognosis in patients with HCC after surgery [39].

m6A2Target database (http://m6a2target.canceromics.org) is the most comprehensive database of target genes corresponding to the three types of m6A enzymes (writers, erasers, and readers) so far. In this database, the validated targets module contains experimentally verified relationship pairs between m6A enzymes and target genes, whereas the Potential Targets module stores the relationship pairs between m6A enzymes and target genes obtained through high-throughput sequencing data analysis. Several studies have confirmed that there was a direct interaction between the m6A enzyme and mRNA using eCLIP-seq [40,41]. However, these results supported by experimental evidence in the database do not include relevant research on HCC. Therefore, further experimental verification is necessary for the correlation between m6A enzyme and target gene in HCC. In conclusion, this study speculated that LINC00665 plays a role in HCC by regulating the above 11 mRNAs via modulation of IGF2BP1, IGF2BP2, and YTHDF1. In so doing, it provided new insights into the roles of lncRNA and m6A enzymes in HCC. Further determination of the regulatory relationship requires more diverse *in vivo* and *in vitro* experiments in the future.
